# Learning semantic similarity from sentence pairs using hybrid features centric approach and explainable siamese neural networks

**DOI:** 10.1371/journal.pone.0345540

**Published:** 2026-05-21

**Authors:** Weihong Zhao, Chunlu Hu

**Affiliations:** Department of Foreign Language and Architecture Engineering, Qingdao Huanghai University, Qingdao, Shandong, China; Dalian Maritime University, CHINA

## Abstract

Semantic embeddings play an important role in modern natural language processing because they help models understand meaning beyond individual words. Accurate text similarity is essential for many applications such as search, automated scoring, summarization, and question answering. However, existing methods based on Term Frequency-Inverse Document Frequency (TF-IDF) or simple lexical overlap often fail when sentences differ in length, structure, or word choice. These methods are less in performance, especially when working with short or medium-length sentences where meaning is expressed in different ways. This study explores sentence-level similarity using a Siamese BiLSTM model that learns deep semantic patterns and relationships between two sentences. The model captures contextual meaning, word interactions, and paraphrastic variations more effectively than traditional approaches. Experimental results show that the proposed model achieves the highest performance among machine-learning regressors, with lower errors and improved stability. Compared to TF-IDF, cosine similarity, and feature-based regressors, the Siamese model provides more accurate judgments of semantic closeness with RMSE of 0.16 and MAE of 0.107. Feature-level analysis using TF-IDF, Jaccard similarity, and embedding distances further supports these findings. Explainable AI techniques (SHAP, LIME) confirm model transparency by highlighting meaningful semantic cues and distributing attention across important linguistic features.

## 1. Introduction

Human communication is built on the ability to understand meaning, even when the same idea is expressed in many ways. A single concept can be described with different words, sentence structures, or levels of detail, yet readers effortlessly grasp the intended message [1]. This significant flexibility makes language rich and expressive, but it also makes computational understanding extremely difficult [2]. As Artificial Intelligence becomes more deeply embedded in everyday applications such as digital assistants, intelligent tutoring systems, content recommendation, and automated writing evaluation [3], the need for machines to interpret meaning with human-like accuracy has become an active and essential area of research [4].

Language plays a central role in this transformation. Modern systems depend on both *Natural Language Generation (NLG)* to produce coherent text and *Natural Language Understanding (NLU)* to interpret user input. Natural Language Processing (NLP) lies at the core of NLU, aiming to model how meaning is constructed, structured, and communicated through grammar, vocabulary, and context [5]. Yet, one of the most difficult tasks in NLP is determining whether two sentences share the same meaning. This requires more than matching words; it requires understanding semantic roles, recognizing patterns, identifying relationships, and interpreting the intent embedded within sentence structure [6].

Understanding how similar two sentences are an important task in natural language processing. People can easily tell when two sentences express the same idea, even if they use different words or grammar [7]. However, teaching machines to make the same judgment is difficult. Modern applications such as search engines, chatbots, learning platforms, recommender systems, and automated scoring depend heavily on accurate text similarity [8]. As language becomes more diverse and creative, the need for models that can understand meaning rather than only match words has become more important [9]. Traditional methods often rely on surface-level signals such as shared words, token overlaps, or simple statistical features [10]. Approaches like TF-IDF, cosine similarity, and n-gram matching work well when sentences are short and very similar. But they fail when meaning is expressed with different vocabulary, sentence length varies, or the structure changes [11]. These limitations highlight a gap between lexical similarity and true semantic understanding. This gap becomes even wider in real-world text where paraphrasing, synonyms, and contextual clues are common. Beyond core NLP tasks, the proposed similarity framework can be effectively applied to sentiment analysis [12,13], enabling complex interpretation of subjective content by comparing emotional tone across texts [14]. In personality detection, it can assess linguistic patterns aligned with psychological traits [15]. Similarly, in mental health analysis, it may aid in identifying semantic shifts or emotional signals indicating stress, anxiety, or depression [16]. These additions, along with applications like essay grading [17], plagiarism detection [18], conversational AI [19], and semantic retrieval [20], highlight the model’s wide-reaching utility in both academic and real-world environments.

The present study explores sentence-level similarity using a Siamese BiLSTM model trained to learn semantic relations between two sentences. The goal is to overcome the weaknesses of traditional similarity measures and to provide a more stable and accurate method for predicting semantic closeness. The model is further compared with machine-learning regressors to highlight improvements in accuracy and consistency. Beyond performance evaluation, this work also includes feature-level analysis and explainable AI techniques which help reveal what the model attends to when judging similarity. Together, these elements show the importance of semantic embeddings and demonstrate why deep models are essential for reliable text similarity prediction.

This study makes several noteworthy contributions to the domain of sentence-level text similarity. First, it explores semantic similarity using contextual embeddings, effectively capturing both syntactic and semantic patterns embedded within labeled sentence pairs. Building upon this, the study integrates lexical and semantic features including cosine similarity, n-gram overlap, sentence length differences, and embedding distance norms (L1 and L2) to provide a comprehensive multi-faceted analysis of sentence similarity. In addition, the research conducts a comparative evaluation of various traditional machine learning models such as Support Vector Regression (SVR), Random Forest, and Gradient Boosting, alongside a TF-IDF baseline, to assess their capacity for modeling sentence similarity. The core innovation lies in the application of a Siamese BiLSTM architecture, designed to align sentence embeddings in a shared space for similarity prediction. This architecture demonstrated superior performance with a mean absolute error (MAE) of 0.107 and root mean squared error (RMSE) of 0.167. Finally, the study incorporates explainable AI techniques, namely SHAP and LIME, to interpret the feature-level contributions toward model predictions, ensuring transparency and interpretability of the predictive framework.

## 2. Related work

Understanding text similarity has become a foundational challenge in natural language processing, particularly in tasks involving sentence-level semantic comparison. Existing research has explored both traditional machine learning and modern deep learning strategies, integrating [21] lexical, statistical, tabular [22] and embedding-based techniques [23]. This section critically examines peer-reviewed studies, also summarizes in Table 1 that investigate how various models quantify similarity using contextual and syntactic signals.

**Table 1:** Compares key aspects of the existing works, including model architecture, embedding techniques, datasets, results, and noted strengths – limitations.

Ref	Year	Model	Technique	Dataset	Results (Metric)	Strength	Limitation	Research Focus
[24]	2026	Bi-LSTM + Attention	Multi-embedding (GloVe, FT, Para)	Quora Question Pairs (DQP)	95.6% Acc, 97.7% F1	Combines multiple embeddings, high accuracy	Task-specific (QA duplicates)	Duplicate question detection (semantic similarity)
[25]	2025	BERT + SVM (Hybrid)	Contextual embed + SVM + attention	Short Texts (Edu answers)	90% Accuracy	Deep semantics via BERT, efficient classification	Limited to short answers domain	Semantic similarity in educational QA grading
[26]	2025	Siamese BERT (SDQKC)	Dynamic QK co-attention + contrast	Terminology definitions (TDS)	r = 0.695 (Pearson)	Novel co-attention, new data generation	Requires synthetic data (GPT-4 pipeline)	Short text similarity in multi-domain terminology
He *et al.* (Zhengfang)	2025	Siamese CNN + Siamese RNN	Two-channel (local+global)	Short texts (NLPCC corpus)	↑ Accuracy vs. single Siamese	Captures local and long-range features	More parameters (dual networks)	Short text semantic similarity (hybrid local/context)
[28]	2022	Re-LSTM (modified LSTM)	Weighted embedding + attention	QQP (Chinese), ATEC (Chinese)	>85% Prec/Rec/Acc/F1	Lightweight LSTM variant, feature gating	Evaluated on Chinese QA only	Text similarity algorithm (weighted RNN)
[29]	2022	BERT + Bi-LSTM (Hybrid)	Fine-tuned BERT + Siamese LSTM	STS Benchmark, MSRP	↑ Accuracy vs. single models	Combines contextual and sequential learning	May overfit if embeddings not well-aligned	General semantic textual similarity
[30]	2022	AraBERT fine-tune + transfer	Translate+fine-tune, ensemble data	Arabic STS 2017 (MSA, dialect)	0.81/0.77 Corr (MSA/dial.)	Overcomes low-resource by TL	Relies on MT quality for training	Arabic semantic textual similarity via transfer
[33]	2025	Random Forest, XGBoost (ML)	Predict sim from embed features	Word similarity (SimLex999)	r = 0.84 (RF Pearson)	Interpretable factors (co-occurrence, etc.)	Focused on word-level (not sentences)	Analyzing embedding similarity factors
[32]	2024	Siamese Bi-LSTM + Attn	Translation augmentation + LSTM	Tibetan–Chinese plagiarisms	↑ Recall vs. baseline	Tackles low-resource plagiarism	Low-resource cross-lingual plagiarism detection	Analyzing only with Chinese data
[34]	2024	Various (TF-IDF, embed, BERT)	Feature engineering + ML/DL grid	Legal cases (BR corpus)	Best: ~ 85% Precision (est.)	Extensive evaluation, granular features	Complex models not always better	Legal precedent retrieval (case similarity)
[21]	2024	CNN + LSTM (Hybrid)	Word2Vec/GloVe embed + dual encoder	Stack Overflow QA pairs	87.1% Acc, 87% Recall	Captures local & global context, fast infer	Moderate accuracy (misses some pairs)	Duplicate tech question identification
[35]	2025	RoBERTa + CHSCSO optimizer	Metaheuristic-enhanced transformer	STS Benchmark, others	> baseline Acc; 0.002s infer	Very fast, avoids overfitting	Added complexity of optimization step	Efficient text similarity via chaotic optimization
[36]	2023	BERT+GCN + Tree Kernel	Knowledge graph + parse integration	STS Benchmark (EN)	0.8805 PCC (STS-B)	Uses semantic + syntactic cues together	High complexity (multiple components)	Short-text similarity with semantic & syntactic fusion
[39]	2022	BERT + Graph U-Net (DGNN)	Dependency+SRL graph with GCN	STS 2017, SICK (EN)	↑ Pearson vs. plain BERT	Incorporates linguistic structure (SRL)	Graph construction overhead	Incorporating SRL knowledge in similarity
[41]	2023	mBERT, XLM-R, etc. (X-lang)	Multilingual transformers compare	USWS-19 (En–Urdu)	Best r ≈ 0.70 (Urdu–Eng)	Handles cross-lingual pairs with no bilingual data	Limited to word-level similarity	Cross-lingual word similarity (English–Urdu)

Recent research has discussed the topic of text similarity with the methods of embedding along with machine and deep learning models. Several pre-trained word embeddings with a Bi-LSTM and attention mechanism. Their joint model also dealt with the issue of class imbalance through resampling based on SMOTE on the Quora question pairs dataset, which was significantly higher than the baseline LSTM models [24]. In a similar way, a BERT+SVM hybrid with multi-level attention in terms of short answer grading. Their model was able to include semantic nuances in student answers using the contextual embeddings of BERT and the effectiveness of classifications on SVM, with the addition of word level attention which is significantly better than the conventional methods [25]. A dynamic query-key co-attention and contrastive learning (SDQKC) Siamese BERT proposed that estimates the term definition similarity. To mitigate data scarcity, they autogenerated a Terminology Definition Similarity corpus and trained their model to obtain Pearson-achieving r of r 0.695 on test sets specific to the domain, showing better performance than baseline models [26]. At capturing the local and long-range features through a two-channel Siamese model, with one branch of CNN capturing the local n-gram features and the other branch of Bi-LSTM capturing the context. This hybrid SCNN-SRNN enhanced similarity in short texts classification because linear models alone had difficulties in local semantics [27].

Contextual embeddings also use transformers, which have dominated such tasks, other scholars explored RNN models to enhance similarity learning. Using Re-LSTM, an adapted LSTM that has two-gate mechanisms and built-in TF-IDF weighting. Incorporating the attention layer with re-weighting of the word feature produced more semantic features of sentence pairs by Re-LSTM [28]. Similarly, fine-tuned BERT embeddings have been used together with a deep Siamese Bi-LSTM classifier. This cross-method, tested on benchmark STS data, was better in semantic similarity detection compared to the use of BERT or Bi-LSTM alone by using the context representation provided by BERT as features to the LSTM model [29]. The study explored the issue by translating and interleaving English STS data to fine-tune Arabic BERT models. They found 0.6 Pearson correlation of Modern Standard Arabic sentence similarity and about 77% on Egyptian and Saudi dialects a significant score as there are just several hundreds of original Arabic pairs [30]. Another study addressed a narrow similarity problem in the patent field. They came up with a group of four Transformer models to fit patent phrases. Their ensemble combining weighted averaging of the various BERT-family models’ outputs with a novel token scoring preprocessor of patent text was found to be better at semantic alignment on the U.S. Patent Phrase-to-Phrase dataset [31]. Siamese networks trained using data augmentation have demonstrated potential in the plagiarism detection field and in low-resource languages. Siamese Bi-LSTM to detect Tibetan Chinese plagiarism, and back-translation was added to it. They had diversified a training set on bilingual training through machine translation, and they had added a pre-detection step through document vector abstractions to be used to filter candidate pairs. The improved Siamese network, which considers the attention mechanism with cross-lingual properties, proved to be more efficient in recalling plagiarized passages in low-resource environments than the base LCS-based methods [32]. The effects of word embeddings are similar in which the author uses machine learning to extract the features of the embeddings. As it is demonstrated through training Random Forest and XGBoost models to forecast human word similarity scores based on statistical variables, found co-occurrence frequency and contextual diversity play a significant role in embedding-based similarity. The highest ML model using human judgments also had r = 0.54 which can be interpreted as to why the embeddings sometimes generate spurious relationships [33].

At the document-level similarity, more than one hundred text representation combinations and algorithms tested on legal case retrieval. Using a Brazilian corpus of court cases, they compared classical vector models, TF-IDF, static embeddings and transformers against several classifiers. They statistically evaluated their methods and found that the ones that were based on the fine-grained text units perform best. Some of the more sophisticated deep models did not consistently provide over and above the results of simpler ones; refinement of textual characteristics could be more effective [34]. A combined bio-inspired optimization and transformers to enhance accuracy as well as the inference speed in similarity evaluation. To ensure that RoBERTa does not descend into local minima, they optimized the embeddings of RoBERTa with a chaotic sand cat swarm optimization algorithm [35]. In addition to plain text, scholars have added linguistic insight to add more to embeddings of similarity. The model suggested by [36] is a composite model, which combines both semantic and syntactic data to do short-text similarity. They refined BERT embeddings with a graph convolutional network trained on external knowledge base and they also computed syntactic similarity on kernels of constituency parse trees [37]. Their model achieved a correlation of 0.68 on STS Benchmark by combining these in a single framework and was superior to recent pure-transformer models [38]. This highlights the importance of the fact that the ability to capture sentence structure and world knowledge can enhance embedding-based similarity. Further integrated semantic role labeling with transformers in a graph neural network process. They transformed the outputs of SRL into a graph and used a Graph U-Net model on top of BERT representations [39]. Transformer-based models of the problem of semantic word similarity in English-Urdu, which is a low-resource cross-lingual task. Based on the USWS-19 benchmark, they compared multilingual, and distillation-based models [40]. The transformers outdid the previous bilingual word embedding methods by a significant margin, particularly when it comes to culturally specific pairs of words, which vindicated the benefit of contextual embedding in multilingual environments [41]. The systematic review of methods of short-text semantic similarity (STSS) has been introduced by [42], they summarize the issues of comprehension of short sentences in the absence of sufficient context and list recent developments in the field of deep learning-based STS.

The latest developments in learning semantic patterns on textual data have demonstrated encouraging outcomes in a wide range of fields with the aid of embedding-based, feature-conscious, and sentence-level representations. SEAD-MGFE-Net [43] uses multigranular feature modeling with adaptive regularization to bring about the aspect-opinion pairings together on complex utterances to refine sentiment analysis in conversations. The contrastive learning and structural calibration in Sci-SpanDet [44] detect AI-generated scientific spans, including stylistic and contextual subtlety outside of the text. HHG-Bot [45] is a high-order graph, which is used to capture user and tweet semantics to efficiently detect bots in noisy social media data. During the same time, ArchSentry [46] identifies semantic structures in the behavior of Android apps through meta-path embeddings and fusion learning to identify malicious examples. Overall, these approaches demonstrate the increased significance of embedding-based and context-sensitive frameworks of semantic processing at different levels in text.

Recent progress in deep learning has shifted the focus toward semantic embeddings that encode meaning in continuous vector spaces. Models like Siamese architecture develop associations among sentence pairs as well as more profound linguistic patterns [47]. The models can identify semantic proximity despite a low degree of lexical similarity. They can detect common intent, decode paraphrased text and can deal with short and medium length sentences. This makes them suitable for more complex NLP tasks that require understanding rather than just matching.

## 3. Proposed research methodology

**Fig 1:**
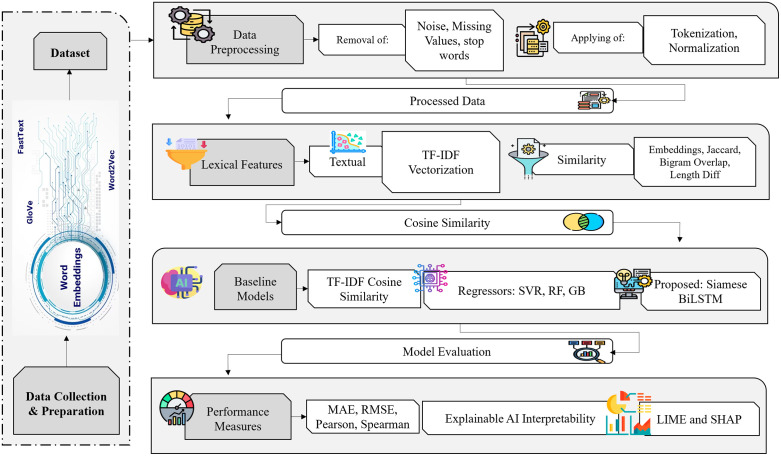
Overall Framework of the Proposed Text Similarity System which begins with text preprocessing and feature extraction, followed till explainability achieved through SHAP and LIME for interpretability..

The proposed methodology is built upon the intuition that semantic similarity cannot be captured by surface features alone; instead, it requires a deeper understanding of sentence structure, contextual meaning, and cross-sentence interactions. Classical lexical or features based approaches cannot generalize over paraphrastic variations and transformer scale models are computationally expensive on medium sized datasets. To close this gap, this study introduces framework as shown in Fig 1, using Siamese BiLSTM model which combines both high quality linguistic embeddings and non-linear interaction modeling. This architecture is the one that does not only encode every sentence independently, but it builds a special similarity space in which semantic relation is explicitly measured, combined, and predicted through a special regression layer. The following subsection presents the complete algorithmic flow and formulation of the proposed model.

### 3.1 Data acquisition and cleaning process

The dataset used in this study is approximately 1.67 MB in size and contains over 13,000 sentence pairs. It was sourced from a public Kaggle repository and is fully anonymized to ensure privacy compliance. Each pair is accompanied by human-annotated similarity scores ranging from 0 to 1, reflecting semantic relatedness. The data is analyzed to comprehend the distribution of similarity scores, sentence length and text patterns. Simple inspection is done to curb data quality such as checking missing values, duplicates or noisy records. This is done to make sure that the dataset is valid and can be used in downstream modeling. Inconsistencies detected during inspection are resolved. Further to ensure cleanliness and usefulness score of dataset, preprocessing is followed which makes the process by analyzing raw sentences ready before they are passed to both the classical and deep learning models. Sentences are purified, that is, they are converted to lowercase, and all unnecessary spaces are eliminated, and some basic normalization is made to guarantee uniformity. The use of tokenization is used to divide sentences into meaningful units [48]. This move guarantees the fact that noise in the text is not used to interfere with feature extraction or learning of neural representations. The lexical, statistical, and embedding-based analyses are carried out on clean and structured text which is the basis of the analysis performed later in the research.

### 3.2 Lexical and similarity features

A group of lexical and statistical similarity characteristics is calculated to assist in supporting baseline modeling and comparative analysis. Each sentence is transformed into TF-IDF vectors and various similarity measurements are derived, i.e., *cosine similarity, Jaccard similarity, length difference and bigram overlap* [49]. Embedding-based difference norms (L1 and L2) are also computed to capture more differences. These characteristics are useful in two functions; they give information about lexical overlapping patterns and provide inputs to the machine-learning regression baselines [50].

To account for lexical frequency and semantic importance, we extend the standard Jaccard index by incorporating token-level weights and frequency-aware matching. This formulation emphasizes informative word overlap while discounting common, less informative terms.


$$\begin{array}{c} {\text{Jaccard}}_{\omega }(S_{1},S_{2})=\frac{\sum_{w\in S_{\text{1}}\cap S_{\text{2}}}\text{min}\left(f_{\text{1}}(w),f_{\text{2}}(w)\right)\cdot \omega_{w}}{\sum_{w\in S_{\text{1}}\cup S_{\text{2}}}\text{max}\left(f_{\text{1}}(w),f_{\text{2}}(w)\right)\cdot \omega_{w}} \end{array}$$ (1)

Where, $S_{1},S_{2}$ token sets of the two sentences, $f_{\text{1}}(w),f_{\text{2}}(w)$ frequency of token win $S_{\text{1}}$and $S_{\text{2}}$, $\omega_{w}$ weight for token w(e.g., IDF or semantic importance). Extends traditional Jaccard by capturing token frequency sensitivity and semantic weight.

To better distinguish structural similarity in sentence fragments, we compute bigram overlap with geometric scaling, allowing finer control over the contribution of short and long n-gram intersections. This smooths overlap intensity and improves sensitivity to partial phrase matches.


$$\begin{array}{c} {\text{BigramOverlap}}_{\gamma }(S_{1},S_{2})=\frac{{\left|B_{\text{1}}\cap B_{\text{2}}\right|}^{\gamma }}{\left({\left|B_{\text{1}}\right|}^{\gamma }+{\left|B_{\text{2}}\right|}^{\gamma }\right)-{\left|B_{\text{1}}\cap B_{\text{2}}\right|}^{\gamma }+\in } \end{array}$$ (2)

Where, $B_{\text{1}},B_{\text{2}}$ bigram sets from $S_{1}$and $S_{2}$,  γ∈(0,1] geometric scaling factor (e.g., 0.5 for root-scaling overlap size), ∈ small constant to prevent zero division. Improves discrimination between sparse vs. dense bigram overlaps with smooth scaling.

### 3.3 TF-IDF cosine similarity

The first baseline is the direct prediction of sentence similarity by the cosine similarity between TF-IDF and GloVe vectors. This method is a traditional lexical matching, in which similarity is determined solely by similar words and frequency of tokens, which computationally analyzed its Weighted Cosine Similarity with Norm Regularization. This model, whilst not very complex, offers a valuable point of reference as to the way in which more significant semantic approaches are more effective than solely lexical alignment.


$$\begin{array}{c} \overset{\omega }{\underset{\text{cos}}{\text{Sim}}}(A,B)=\frac{\sum_{i=\text{1}}^{n}\omega_{i}\cdot a_{i}\cdot b_{i}}{\sqrt{\sum_{i=\text{1}}^{n}\omega_{i}\cdot \overset{\text{2}}{\underset{i}{a}}+\in }\cdot \sqrt{\sum_{i=\text{1}}^{n}\omega_{i}\cdot \overset{\text{2}}{\underset{i}{b}}+\in }} \end{array}$$ (3)

Where, $A=[a_{\text{1}},a_{\text{2}},\ldots ,a_{n}]$ and $B=[b_{\text{1}},b_{\text{2}},\ldots ,b_{n}]$ are embedding vectors of the two sentences, $\omega_{i}$ is a learned or static importance weight for dimension i(e.g., TF-IDF, attention, or IDF). ∈ is a small constant (e.g., ${\text{1}\text{0}}^{-\text{8}}$) to prevent division by zero and stabilize gradients in deep models. This similarity not only computes directionality but also emphasizes semantically dominant dimensions via ω, and is regularized for numerical robustness.

### 3.4 Baseline models with machine-learning regressors

Regression models are trained on the engineered features in the second baseline including SVR (RBF kernel), Random Forest, and Gradient Boosting [51]. These models are trained in the relationship between measures of lexical similarity and ground-truth measures of similarity. Their performance assists in setting the contribution of the handcrafted features and shows their limitation, particularly with the need to form sentences with similar meaning but different structure or vocabulary.

### 3.5 Proposed siamese bidirectional long short-term memory

The main part of the study presents a Siamese BiLSTM framework that is learned to acquire deep semantics between sentence pairs. The model takes two branches of BiLSTM with the same weights to produce contextual embeddings of every sentence. These embeddings are word interactions, sequence patterns and semantic roles [52]. The two embeddings are separated by their distance which is predicted by the dense layers, which depends on hyperparameter settings displayed in Table 2. The Smooth L1 loss is used to deal with uncertainty in human similarity scores and achieve stable learning. The algorithm presented in the proposal proposes a Siamese BiLSTM architecture that is aimed to represent semantic similarity through the concomitant learning of contextual, structural, and interaction-level relationships between pairs of sentences [53]. The approach incorporates the use of multi-channel token embeddings, bi-directional contextual encoding, semantic distance metrics and interaction operators into a single similarity space.

**Table 2:** Hyperparameter Settings for the Proposed Siamese BiLSTM Model.

Parameter	Value
Embedding dimension	300
Embedding type	Trainable word embeddings
Initialization	Xavier uniform
Vocabulary size	Based on dataset tokenizer
LSTM hidden size	128 units per direction
Number of BiLSTM layers	1
Dropout (recurrent/output/overall)	Recurrent: 0.20, Output: 0.30, Overall: 0.3
Weight sharing	Shared encoder for both sentences
Distance metric	Absolute difference, L1 + L2 norms
Feature combination	Concatenation
Dense layer size	128 → 64 → 32
Activation function	ReLU
Output activation	Linear
Batch normalization	Applied in dense layers
Final output	Single similarity score (0–1)
Loss function	Smooth L1 (Huber) Loss
Optimizer	Adam
Learning rate	0.001
Weight decay	1e-5
Batch size	64
Number of epochs	20–30
Early stopping	Patience = 5
Gradient clipping	Max-norm = 5
L2 regularization	1e-5

### Algorithmic flow of the proposed siamese BiLSTM semantic similarity model

**Goal:** Predict continuous similarity score $\hat{y}\in [\text{0},\text{1}]$ for a sentence pair $\left(S_{\text{1}},S_{\text{2}}\right)$.

#### Step 1. Text Preprocessing and Token–Level Encoding.

We represent each sentence S as a structured sequence that combines tokens, part-of-speech tags, and length cues,


$$\begin{array}{c} S=\{(w_{t},p_{t},{\text{??}}_{t})\overset{n}{\underset{t=\text{1}}{\}}} \end{array}$$ (4)

These units are jointly projected into an embedding manifold by a composition operator $\Phi_{\text{emb}}$,


$$\begin{array}{c} E=\Phi_{\text{emb}}(S)=[f_{w}(w_{t})\;\;∥\;\;f_{p}(p_{t})\;\;∥\;\;f_{\text{??}}({\text{??}}_{t})\overset{n}{\underset{t=\text{1}}{]}}\;\;\overset{\;\;\Pi \;}{\to }\;\;R^{n\times d} \end{array}$$ (5)

where Π aligns heterogeneous linguistic signals $\{w_{t},p_{t},{\text{??}}_{t}\}\to$unified semantic vectors.

#### Step 2. Bidirectional Semantic Accumulation (BiLSTM Encoder).

The embedding sequence E is encoded through a dual directional flow that aggregates left and right context,


$$\begin{array}{c} E\overset{\;\;\text{BiLSTM}\;}{\to }H=\{h_{t}\overset{n}{\underset{t=\text{1}}{\}}} \end{array}$$ (6)

Each contextual state $h_{t}$ is generated by a coupled update that fuses past (→) and future (←) information,


$$\begin{array}{c} h_{t}=\Psi \left({\vec{h}}_{t-\text{1}},e_{t},{\overset{\leftarrow }{h}}_{t+\text{1}}\right)=\sigma \left(W_{f}e_{t}+U_{f}{\vec{h}}_{t-\text{1}}+U_{b}{\overset{\leftarrow }{h}}_{t+\text{1}}+b\right) \end{array}$$ (7)

so every position is influenced by past → present ← future context.

#### Step 3. Global Sentence Representation Extraction.

Contextual states $H=\{h_{t}\}$ are condensed into a single sentence embedding by a competitive pooling–plus–attention operator,


$$\begin{array}{c} H\overset{\;\;\Gamma \;\;}{\to }h \end{array}$$ (8)

where Γ(·) refines max pooling with soft relevance weighting,


$$\begin{array}{c} h=\Gamma (H)=\underset{t}{\text{max}}\;(h_{t})\;\;+\;\;\alpha \;\text{SoftMax}(W_{s}H) \end{array}$$ (9)

Thus each sentence yields a compact representation.


$$\begin{array}{c} S_{\text{1}}\mapsto h_{\text{1}},\;\;S_{\text{2}}\mapsto h_{\text{2}} \end{array}$$ (10)

#### Step 4. Semantic Interaction and Distance Modeling.

Pairwise semantic relations are captured by combining symmetric distances and directional interactions [54],


$$\begin{array}{c} \left(h_{\text{1}},h_{\text{2}})\overset{\;\;\Lambda \;\;}{\to }(d_{\text{L1}},d_{\text{L2}},d_{\text{cos}},d_{⊙}\right) \end{array}$$ (11)

where the interaction operator Λ is defined as


$$\begin{array}{c} \Lambda (h_{\text{1}},h_{\text{2}})=\{\begin{array}{c} d_{\text{L1}}=∥h_{\text{1}}-h_{\text{2}}∥,\\ d_{\text{L2}}=∥h_{\text{1}}-h_{\text{2}}{∥}_{\text{2}}\text{1},\\ d_{\text{cos}}=\frac{\overset{⊤}{\underset{\text{1}}{h}}h_{\text{2}}}{∥h_{\text{1}}∥∥h_{\text{2}}∥}\text{1},\\ d_{⊙}=h_{\text{1}}⊙h_{\text{2}}, \end{array} \end{array}$$ (12)

and 1 denotes broadcasting to match the embedding dimension.

#### Step 5. Pair Representation in Similarity Space.

All interaction components are fused into a single similarity-space vector $z_{\text{pair}}$,


$$\begin{array}{c} (d_{\text{L1}},d_{\text{L2}},d_{\text{cos}},d_{⊙})\overset{\;\;\text{concat}\;\;}{\to }z_{\text{pair}} \end{array}$$ (13)

with the fusion defined as


$$\begin{array}{c} z_{\text{pair}}=\left[h_{\text{1}}\;\;∥\;\;h_{\text{2}}\;\;∥\;\;d_{\text{L1}}\;\;∥\;\;d_{\text{L2}}\;\;∥\;\;d_{\text{cos}}\;\;∥\;\;d_{⊙}\right]\in R^{k} \end{array}$$ (14)

This vector encodes both what is shared and what is different between the two sentences.

#### Step 6. Nonlinear Similarity Projection (Regression Head).

The similarity-space representation is projected into a latent similarity manifold through a nonlinear transformation,


$$\begin{array}{c} z_{\text{pair}}\overset{\;\;f_{\text{sim}}\;\;}{\to }z_{\text{sim}} \end{array}$$ (15)

where


$$\begin{array}{c} z_{\text{sim}}=f_{\text{sim}}(z_{\text{pair}})=\varphi (W_{\text{2}}\;\varphi (W_{\text{1}}z_{\text{pair}}+b_{\text{1}})+b_{\text{2}}) \end{array}$$ (16)

and φ(·) is a pointwise activation (e.g., ReLU) shaping higher-order similarity patterns.

#### Step 7. Similarity Score Generation.

A final linear projection maps the latent similarity vector to a scalar score in [0,1],


$$\begin{array}{c} z_{\text{sim}}\overset{\;\;\omega \;}{\to }\hat{y} \end{array}$$ (17)

with


$$\begin{array}{c} \hat{y}=\omega (z_{\text{sim}})=\sigma_{\text{reg}}(w^{⊤}z_{\text{sim}}+b_{\text{out}}) \end{array}$$ (18)

where $\sigma_{\text{reg}}$ is a bounded regression activation (e.g., sigmoid) ensuring valid similarity range.

#### Step 8. Training Objective and Parameter Update.

For a gold similarity score y, the model is trained by minimizing a Smooth L1 (Huber) regression objective,


$$\begin{array}{c} (y,\hat{y})\overset{\;\;L\;}{\to }L_{\text{Huber}} \end{array}$$ (19)

defined as


$$\begin{array}{c} L_{\text{Huber}}(y,\hat{y})=\{\begin{array}{cc} \frac{\text{1}}{\text{2}}(y-\hat{y}{)}^{\text{2}}, & \left|y-\hat{y}\right|<\delta ,\\ \delta \left|y-\hat{y}\right|-\frac{\text{1}}{\text{2}}\delta^{\text{2}}, & \text{otherwise}, \end{array} \end{array}$$ (20)

and parameters are updated via gradient descent,


$$\begin{array}{c} \theta \leftarrow \theta -\eta \;\nabla_{\theta }L_{\text{Huber}}(y,\hat{y}) \end{array}$$ (21)

#### Step 9: Outcome.


$$\begin{array}{c} (S_{\text{1}},S_{\text{2}})\;\;\overset{\Phi_{\text{emb}}}{\to }\;\;E_{\text{1}},E_{\text{2}}\;\;\overset{\text{BiLSTM}}{\to }\;\;h_{\text{1}},h_{\text{2}}\;\;\overset{\Lambda }{\to }\;\;z_{\text{pair}}\;\;\overset{f_{\text{sim}}}{\to }\;\;z_{\text{sim}}\;\;\overset{\omega }{\to }\;\;\hat{y} \end{array}$$ (22)

**Fig 2:**
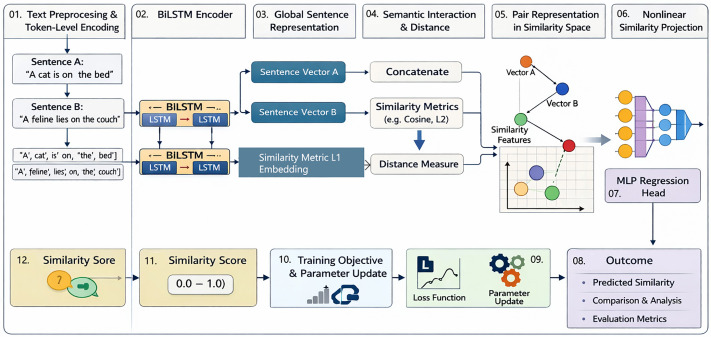
Logical flow diagram of the proposed Siamese BiLSTM-based semantic similarity framework, illustrating each processing step from initial token-level text encoding to final similarity score generation, including semantic accumulation, sentence interaction modeling, and nonlinear projection through a regression head.

The model learns to make judgments of similarity akin to those of humans, by means of this hierarchical flow, between token-level representation and pair-wise semantic fusion. These representations are further refined by the regression head to produce a continuous similarity score that is trained using Smooth L1 loss, which is stable to optimize and predictive behavior on a variety of sentence lengths and patterns of language. The proposed Siamese BiLSTM framework operates through a structured multi-step pipeline. It begins with token-level preprocessing and encoding, followed by bidirectional contextualization using BiLSTM layers. Sentence-level representations are then compared via semantic distance metrics and passed through a nonlinear regression head to predict similarity scores. The full process is summarized in the logical flow shown in Fig 2.

### 3.6 Performance evaluation metrics

The results of the Siamese BiLSTM model are tested on unknown test sets. Regression measures of standard similarity, such as MAE, RMSE, Pearson correlation and Spearman rank correlation are calculated to measure accuracy, consistency, and ability to rank [55]. To evaluate the effectiveness of both baseline and proposed models, we employ multiple statistical metrics that capture prediction accuracy and correlation between predicted and actual similarity scores.

#### 3.6.1 Mean absolute error (MAE).

Measures the average absolute difference between predicted (${\hat{y}}_{i}$) and true similarity values ($y_{i}$).


$$\begin{array}{c} \text{MAE}=\frac{\text{1}}{n}\sum_{i=\text{1}}^{n}\left|y_{i}-{\hat{y}}_{i}\right| \end{array}$$ (23)

#### 3.6.2 Root Mean Squared Error (RMSE).

Emphasizes larger errors by squaring the residuals before averaging.


$$\begin{array}{c} \text{RMSE}=\sqrt{\frac{\text{1}}{n}\sum_{i=\text{1}}^{n}{\left(y_{i}-{\hat{y}}_{i}\right)}^{\text{2}}} \end{array}$$ (24)

#### 3.6.3 Pearson Correlation Coefficient (ρ).

Assesses the linear relationship between predicted and actual similarity scores.


$$\begin{array}{c} \rho =\frac{\sum_{i=\text{1}}^{n}(y_{i}-\bar{y})({\hat{y}}_{i}-\bar{\hat{y}})}{\sqrt{\sum_{i=\text{1}}^{n}(y_{i}-\bar{y}{)}^{\text{2}}}\cdot \sqrt{\sum_{i=\text{1}}^{n}({\hat{y}}_{i}-\bar{\hat{y}}{)}^{\text{2}}}} \end{array}$$ (25)

#### 3.6.4 Spearman Rank Correlation (ρₛ).

Evaluates monotonic relationships using ranked variables, less sensitive to outliers.


$$\begin{array}{c} \rho_{s}=\text{1}-\frac{\text{6}\sum_{i=\text{1}}^{n}\overset{\text{2}}{\underset{i}{d}}}{n(n^{\text{2}}-\text{1})}\text{where }d_{i}=\text{rank}(y_{i})-\text{rank}({\hat{y}}_{i}) \end{array}$$ (26)

The findings are compared to all baseline models which prove the efficacy of deep semantic embeddings to convey the sense beyond the lexical overlap.

### 3.7 Implementation details

The research design of the experiment was to guarantee performance as well as reproducibility in real-life situations. A Tesla P100 graphics board with 16 GB of RAM and a fast CPU made it possible to train deep models, such as the Siamese BiLSTM architecture, as reported in Table 3. A balanced evaluation was in place as the dataset was meticulously divided into training, validation and tests portions in a 70:15:15:15 ratio. TF-IDF and GloVe embeddings were combined to ensure that lexical and semantic features were obtained. The standard metrics applied along with the MAE, RMSE, Pearson and Spearman correlations made this possible as the models were benchmarked in a similar fashion. Secondly, the use of explainability algorithms such as SHAP and LIME brought interpretability to the choices of the models, which strengthens the clarity of the proposed method.

**Table 3:** Experimental Setup and Implementation Details.

Component	Specification
Hardware	
GPU	NVIDIA Tesla P100 (16 GB VRAM)
CPU	Intel Xeon @ 2.3 GHz
RAM	32 GB DDR4
Software Environment	
Operating System	Ubuntu 20.04 LTS (Kaggle Notebook Runtime)
Programming Language	Python 3.10
Deep Learning Framework	PyTorch 2.x
Additional Libraries	scikit-learn, NumPy, Matplotlib, SHAP, LIME
Dataset Configuration	
Total Sentence Pairs	13,000 (approx.)
Train/Validation/Test Split	70%/ 15%/ 15%
Input Representation	TF-IDF vectors, GloVe embeddings (300-dim)
Evaluation Metrics	MAE, RMSE, Pearson ρ, Spearman ρₛ
XAI Tools Used	SHAP (TreeExplainer), LIME (TabularExplainer)

## 4. Results and discussion

The results of the baseline show evident performance levels of the traditional similarity estimation methods. It is demonstrated that the TF-IDF cosine model has the lowest performance, maximum MAE (0.197), RMSE (0.252), and comparatively low Pearson (0.486) and Spearman (0.396) correlations. The TF-IDF is entirely lexical and does not reproduce semantic overlaps, contextual paraphrasing, and word-order effects. As a result, the model usually errs by perceiving similarity in differences in the surface structures but the similarity in the meaning of those sentences, resulting in significant prediction errors and low rank correlations. Application of machine-learning regressors can lead to much higher performance, as results shown in Table 4. The RBF kernel SVR minimizes the MAE at 0.131 and both high Pearson and Spearman a (0.735 and 0.718) correlations. This shows that the RBF kernel has the ability of capturing nonlinear relationships between manually crafted lexical features and similarity scores. Random Forest has a slightly lower MAE (0.126) and slightly lower correlations than SVR. Implying that it is more sensitive to interactions between features, but less sensitive to monotonic ranking patterns than kernel methods. Gradient Boosting also does the same, as the minimum RMSE between baselines is (0.173) and the highest baseline correlations. Overall, the baseline findings suggest that although feature-based regressors can address the weaknesses of TF-IDF. They are limited to lexical representations and are unable to acquire semantic abstraction completely. Siamese BiLSTM model has the best performance on all metrics, and it is evidently performing better than the classical baselines. It achieves the minimum MAE (0.107) and RMSE (0.167) which is a significant error decrease over TF-IDF and significant beneficial change over SVR, RF, and GBDT. More importantly, the model has the highest Pearson (0.767) and Spearman (0.751) values, which means that it does not only predict similarity values more accurately. Moreover, it maintains the relative order of similarity scores in a more constant way. This indicates a more profound and solid conceptualization of semantic equivalence in the pair of sentences. The statistical tests further shed more light on the importance and reliability of the performance of each model. According to the findings, the evaluation of models was done based on paired t-test and Wilcoxon signed-rank tests with a significance level of p = 0.05. Siamese BiLSTM model is the only one that shows a very significant Wilcoxon value (22.00) that shows a strong alignment with ground truth rankings and shows lowest negative t-test value (−0.88), which implies little directional bias. Conversely, such baseline models as TF-IDF cosine (t = −13.62, W = −2.91), SVR (t = −5.12, W = −2.38), Random Forest (t = −5.87, W = −2.51), and Gradient Boosting (t = −4.83, W = −2.42) have moderate to low significance, with stronger deviations in both measures. Such negative values suggest systematic under- or over-prediction effects, and little consistency with human rated similarity scores. All in all, the statistical significance level is fulfilled by means of the BiLSTM model, but at the same time, the semantic structure is more accurately represented, which supports the idea of the reliability of the sentence similarity estimation by this model.

**Table 4:** Comprehensive results analysis of all applied models for text similarity.

Model	MAE	RMSE	Pearson	Spearman	t-test	Wilcoxon
TFIDF cosine [50]	0.197	0.252	0.486	0.396	−13.62	−2.91
SVR [52]	0.131	0.177	0.735	0.718	−5.12	−2.38
RF [51]	0.126	0.188	0.708	0.710	−5.87	−2.51
GB [31]	0.132	0.173	0.747	0.727	−4.83	−2.42
Siamese BiLSTM	**0.107**	**0.167**	**0.767**	**0.751**	**−0.88**	**22.0**

This is since the performance of the Siamese BiLSTM is enhanced by the encoding of contextual word dependencies, semantic structure, and paraphrastic variations using trainable embeddings and bidirectional recurrent encoding. The BiLSTM, unlike TF-IDF or hand-crafted features, also captures meaning when sentences with minimal similarities in common words appear which allows the modelling of lexical and semantic similarity effectively. The findings affirm that deep contextual embeddings are much better in estimating semantic similarity and give a much better and more linguistically inspired interpretation than the classical shallow feature-based models.

**Fig 3:**
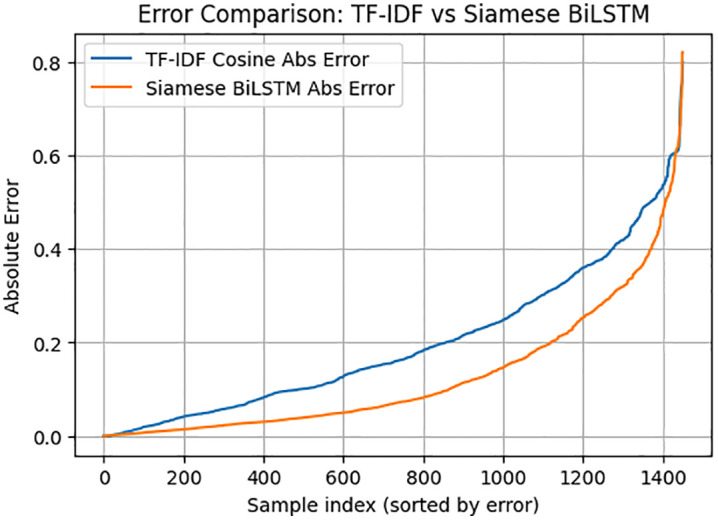
Error Comparison Between TF-IDF Cosine Baseline and Siamese BiLSTM Model.

The comparison of the performance of the classical TF-IDF and the proposed Siamese BiLSTM model shows that deep semantic representations obviously outperform the classical one. Fig 3 demonstrates that the absolute error curves of the two models indicate a consistent difference in performance with the Siamese BiLSTM having significantly lower error in almost the whole range of samples. TF-IDF model increases in error by a significant margin on medium and high difficulty samples whereas the Siamese model has a smoother and more controlled error curve. This finding underscores the potential of the BiLSTM to recognize semantic equivalence even though sentences differ markedly in the wording, length or structure, which static lexical models cannot surmount.

**Fig 4:**
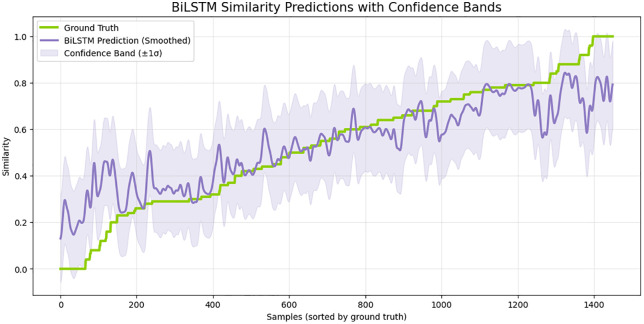
Analysis of BiLSTM Similarity Predictions with Confidence Bands.

More indicators of the strength of the model emerge in the confidence-band analysis presented in Fig 4. In this case, the smoothed BiLSTM predictions are close to the ground-truth similarity trend, as well as the + /-1 sigma confidence band follows a consistent narrow band across most of the similarity spectrum. The narrow confidence zone indicates consistent learning and low variance of predictive behavior particularly in the mid-range similarity levels where traditional models tend to fail. The model is reasonably faithful in making the distinction between low-, medium- and high-similarity pairs and this indicates that the model has good knowledge of semantic progression.

**Fig 5:**
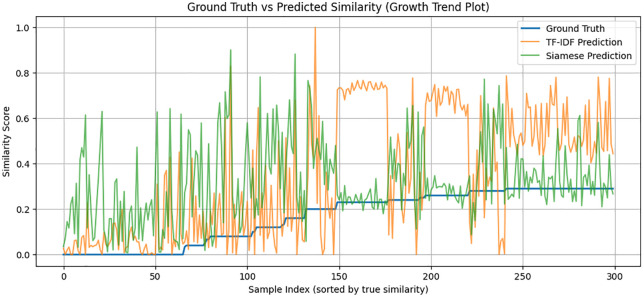
Ground Truth vs. Predicted Similarity: TF-IDF vs. Siamese BiLSTM.

**Fig 6:**
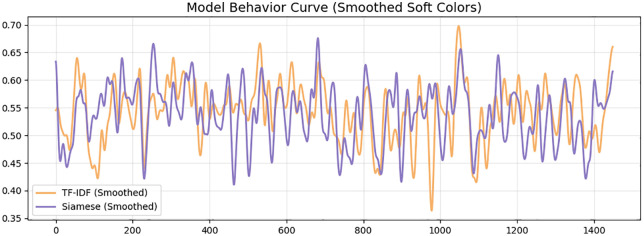
Smoothed output curves for TF-IDF and Siamese BiLSTM predictions exhibit lower variance and more stable behavior, indicating stronger semantic representation and reduced sensitivity to lexical noise.

**Fig 7:**
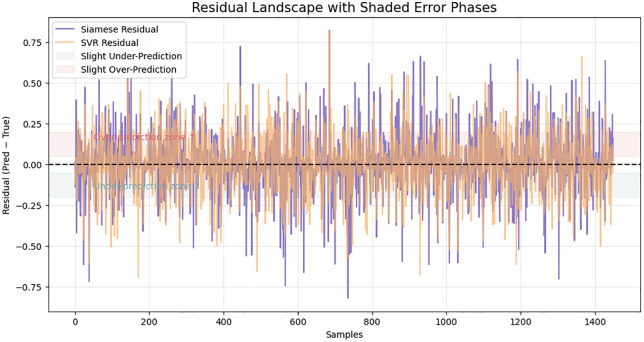
Residuals prediction analysis of the Siamese BiLSTM compared to the SVR baseline.

**Fig 8:**
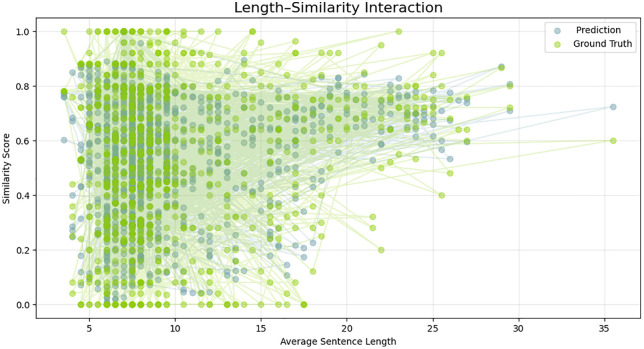
The relationship between average sentence length and the corresponding similarity scores for both the predicted values (light blue) and the ground truth annotations (green).

This behavior is further highlighted in the growth trends comparison in Fig 5. Though the TF-IDF model shows unstable and fluctuating predictions particularly in cases involving moderately similar pairs, the Siamese one gives a more consistent and monotonic curve that fits the ground truth more. The swinging oscillations of the TF-IDF result indicate that the model is dependent on the overlap of tokens on the surface, but the deep embedding model has a higher potential for generalization between paraphrased or lexically distant pairs of sentences. This establishes the fact that the Siamese encoder manages to abstract sense beyond syntactic similarity. Fig 6 further develops the behavioral perspective in a deeper manner by showing smoothed model curves that show how the two models respond to the complexity of some samples. The Siamese curve is always placed in a tighter band, and it is not as fluctuating as TF-IDF. This fluency is indicative of the power of distributed semantic representations, which create similar gradients of continuous, steady-like form, not the token-like sparse ones. Consequently, the similar transitions of the Siamese model are more reliable, which makes it easier to interpret and use downstream. Fig 7 shows an even more detailed picture with residual-level diagnostics. The residuals of the Siamese model are close to zero and it has fewer extreme outliers compared to the SVR baseline. The colored areas reveal that the BiLSTM has a balanced behavior of not biased towards either being an under- or over-predictor of the classical models that often have the tendency to be biased towards one side. The smaller residual variance justifies the capability of the model to encompass finer semantic associations. Furthermore, Fig 8 each point represents a sentence pair, while connecting lines highlight discrepancies in similar judgments across varying lengths. The cluster density on shorter lengths suggests greater prediction confidence, whereas wider dispersion in longer sentences indicates increased variance and challenge in capturing semantic similarity as sentence complexity grows.

**Fig 9:**
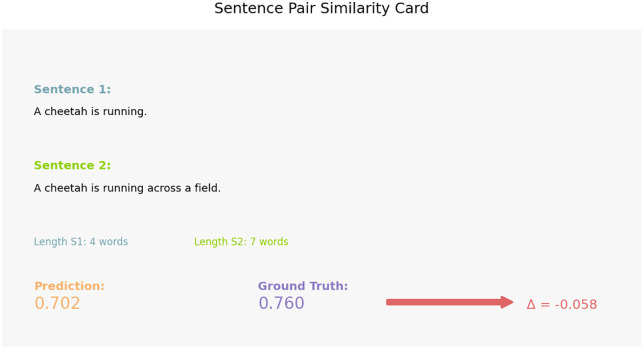
BiLSTM prediction compared to ground truth, along with sentence lengths and prediction error (Δ). The near-zero error demonstrates accurate semantic understanding even with differences in sentence structure or length.

**Fig 10:**
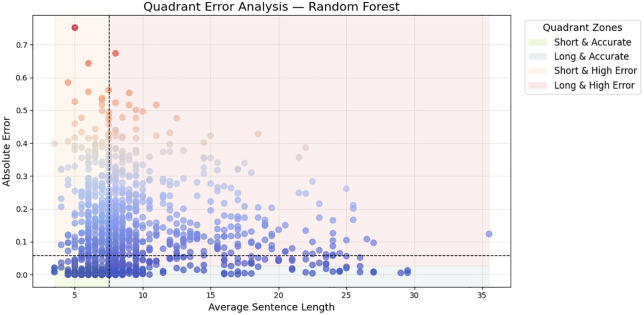
Analysis of absolute error as a function of average sentence length, divided into four interpretative quadrants shows Siamese model performs strongly in both accurate quadrants, showing robustness to sentence length variation.

**Fig 11:**
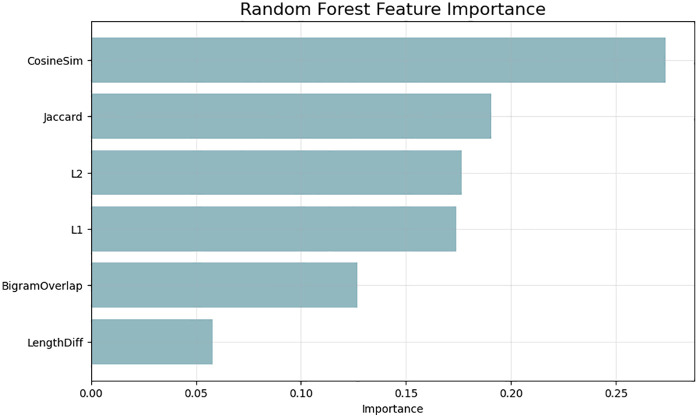
The feature-importance plot shows the relative contribution of lexical and embedding-based features.

Sentence level interpretability Fig 9 is one such example of an individual prediction in the form of a natural-language similarity card. The slight inconsistency between the estimated (0.702) and actual (0.760) similarity follows to show that the model is effective at determining the semantic similarity between two paraphrased sentences, although the sentence length in one sentence varies. The example supports the idea that the Siamese architecture ensures the semantic fidelity of the granular level, resulting in the context-dependent predictions that are highly close to human judgments. The correlation between the length of a sentence and error is developed in Fig 10 where prediction behavior is categorized in quadrant regions. Siamese model is most effective in the Short and accurate and Long and accurate zones where it is very resistant to error inflation even when dealing with longer and structurally richer sentences. Even though there are several high-error points on the Short and High Error quadrant there are comparatively few, which implies that complex semantic nuances rather than length contribute to the fact that there are remaining mispredictions. Lastly, Fig 11 shows the results of feature-importance of the proposed model to put the benefits of the deep model into perspective. Classical features contributions are dominated by similarity on cosine, and similarity on Jaccard, but deep embeddings implicitly represent finer semantic dimensions (synonyms, paraphrase structure, context relationships, etc.). The relatively less significance of length difference and n-gram overlap presents the weaknesses of shallow lexical characterizations and emphasizes the reasons that semantic neural characterizations are superior to handcrafted features.

Altogether, the numbers show that the Siamese BiLSTM presents a significant improvement in semantic comprehension. It minimizes error of prediction, normalizes similarity curves, generates narrow confidence bands and fewer deviations of residues. It is especially strong with paraphrased sentences and lexically diverse constructions- which is a source of failure with traditional models. The model is validated by the visual analyses as internalizing deep semantic structure and is therefore appropriate in semantic similarity tasks in the real world which require contextual based reasoning and not token based matching.

### 4.1 Feature-level analysis for the proposed semantic model

The statistical overview of the metrics of the feature presented in Table 5 forms a valuable background to the interpretation of the idea of semantic similarity in the context of the sentence pairs and the proposed Siamese BiLSTM model. The values of cosine similarity and Jaccard similarity are moderately high with reference to a considerable number of samples indicating existence of lexical-level overlap between a pair of sentences. Nevertheless, embedding-based L1-L2 distances are also highly varied, which supports the fact that there are deeper semantic differences in addition to surface lexical similarity. The existence of high-overlap and low-overlap samples shows the necessity to have a model that could reason on the various levels of linguistics. This is consistent with the reason of using a Siamese BiLSTM, which has the ability to extract both syntactic and semantic indicators which the traditional lexical features may not be able to capture comprehensively.

**Table 5:** Statistical numerical results analysis of semantic similarity metrics for individual sentence pairs.

Index	Cosine Similarity	Jaccard Similarity	Length Difference	Bigram Overlap	Embedding L1	Embedding L2
0	0.392039	0.750000	2	0.000000	22.618063	3.030118
1	0.561629	0.416667	7	0.307692	23.989744	3.019403
2	0.528814	0.750000	3	0.444444	7.585757	0.930071
3	0.305860	0.230769	0	0.076923	5.846624	0.696706
4	0.842867	0.521739	4	0.384615	18.000841	2.087084
5	0.735830	0.571429	1	0.500000	9.445513	1.245832
6	0.723074	0.555556	4	0.272727	13.106157	1.398854
7	0.623421	0.454545	7	0.307692	20.636702	2.384700
8	0.120852	0.083333	1	0.000000	14.084520	1.577327
9	0.835405	0.857143	1	0.571429	3.579847	0.412452

**Fig 12:**
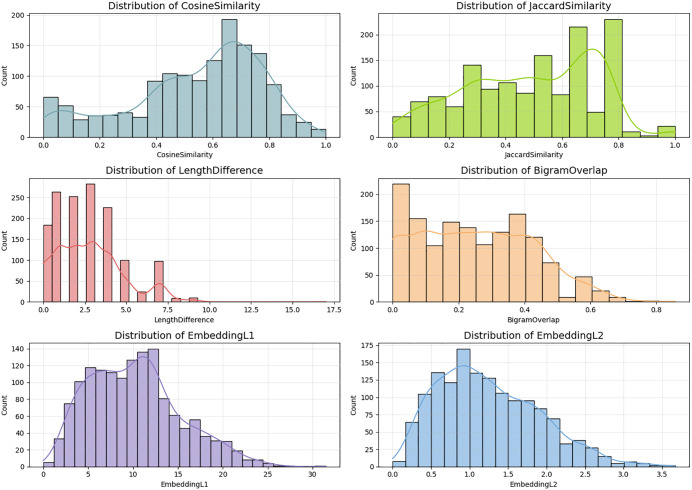
Distribution Analysis of Feature Metrics shows the skewed patterns, and lexical metrics with multi-modal behavior, reflecting the dataset’s varied semantic relationships.

The additional features spread in the dataset are further explained in the distribution plots in Fig 12. Cosine similarity and Jaccard similarity exhibit multi-modal behaviour which implies that there are several groups of relationships between sentence-pairs fully aligned paraphrases, semi-similar pairs, and dissimilar pairs. In comparison, embedding L1 and L2 distances are distributed in smoother right-skewed distributions, which is a result of the continuous semantic space that the encoder generates. The bell-shaped distribution of the embedding features is smoother which indicates that the Semantic gradients generated by the Siamese network are meaningful and can be applied in the model to capture the minute differences in meaning. The length difference distribution also has indicated that by far majority of sentences have a difference of few words but there is a long tail of greater differences; this is the reason why lexical models are not resilient to length imbalance and BiLSTM is more resilient to their imbalance.

**Fig 13:**
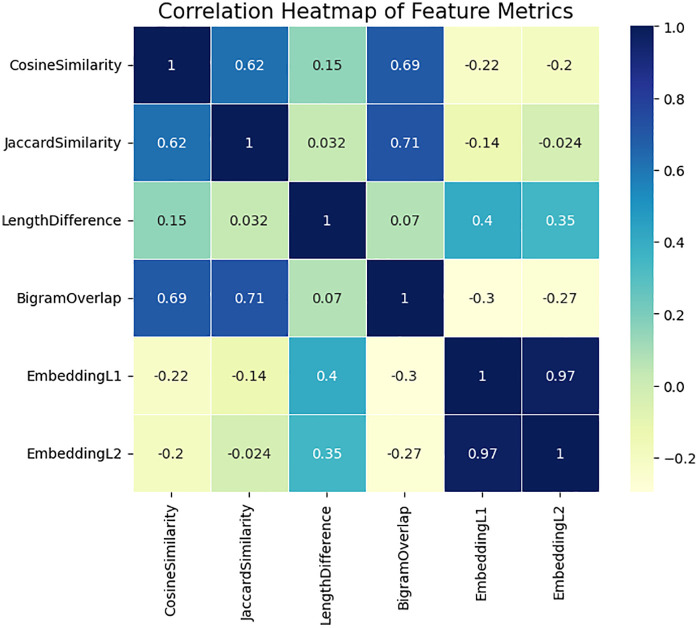
Correlation structure analysis among lexical similarity measures and embedding-based distances.

Fig 13 corrhelogram gave a better understanding of which features are most closely associated with semantic similarity. Cosine Similarity and Jaccard Similarity lexical characteristics are highly and moderately correlated with each other (0.62 and 0.69 and 0.71) and with Bigram Overlap, respectively, which supports the notion that lexical similarity measures represent overlapping structural information. The fact that they are weakly related to embedding distances (negative or very low positive r values between 0.40 and −0.22) however, indicates that embedding-based features encode an alternate dimension of meaning the one that reflects semantic subtlety and not semantic surface overlap. The high correlation between Embedding L1 and Embedding L2 (0.97) supports the fact that Embedding L1 and Embedding L2 are used as the measurement of the same underlying semantic distance with different geometrical interpretations. This distinction between lexical similarity and embedding similarity proves the strengths of the proposed model: lexical measures provide a localized token-based correspondence, whereas the BiLSTM embeddings provide contextual, syntactic, and semantic correspondence that extends well beyond lexical correspondence.

In general, the analysis at the feature level justifies the reasons why the Siamese BiLSTM model is better than the traditional TF-IDF and regression-based baselines. The data is a heterogeneous combination of sentence-pair relations, some of which are lexically similar but semantically distant, others lexically different but semantically similar. The classical metrics do not work in such conditions of cross signal (they depend too much on obtaining the tokens overlap). Conversely, the embedding distributions and the weak dependence on lexical features show that deep representations obtain latent meaning that is beyond the ability of n-gram overlap and length ratios to obtain. This multi-view feature landscape is the reason why the proposed semantic model will yield lower errors and more consistent predictions with misleading lexical cues or sparse lexical cues.

### Interpretability analysis for the proposed semantic model

**Fig 14:**
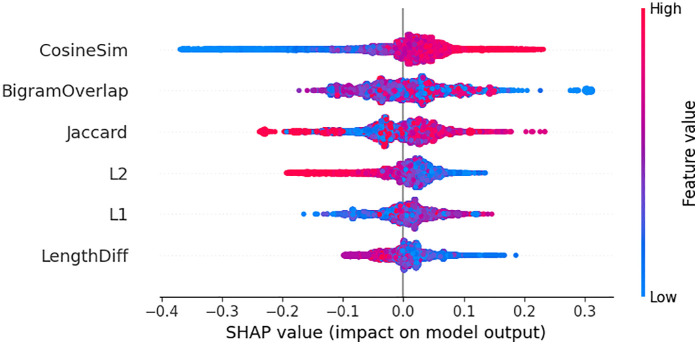
Global SHAP values showing the importance and direction of each feature in the model.

The SHAP summary plot in Fig 14 shows the way that each of the engineered similarity features performs with the model according to the entire dataset. Cosine similarity has the most persistent and significant positive effect, which proves that sentence-level directional alignment is the most significant signal of similarity prediction. Similarity of Jaccard and L2 distance also show significant positive effects that reveal that the model is based on the overlap of lexicon and the proximity of embedding space. Length Difference and Bigram Overlap, in contrast, show mixed effects, with both positive and negative SHAP values; indicating that these two features are conditionally useful, i.e., they are useful with a particular structure but induce noise when sentences vary significantly in length or phrasing.

**Fig 15:**
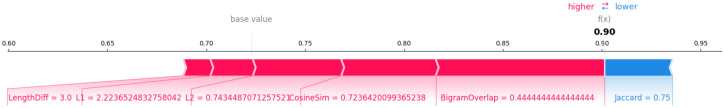
SHAP Force Plot for Local Similarity Explanation showing how individual similarity features push the model’s prediction higher (red) or lower (blue) relative to the base value.

In Fig 15, the SHAP force plot indicates the contribution of each of the engineered similarity features to the final predicted similarity score of a particular sentence pair. Attributes like Length Difference, L1 distance, L2 distance, Cosine Similarity and Bigram Overlap mostly drag the prediction towards the upward direction (red) which means that the values of the attributes will strongly favor an increase in the similarity score. On the other hand, Jaccard similarity has a downward pull (blue direction) indicating that the lexical set-overlap between the two sentences is not as strong as other semantic cues. The graphic left to right flow easily depicts the cumulative movement of the output of the model base value to the ultimate predicted score of 0.90 which offers a clear and intuitive explanation of the local decision-making process of the model.

**Fig 16:**
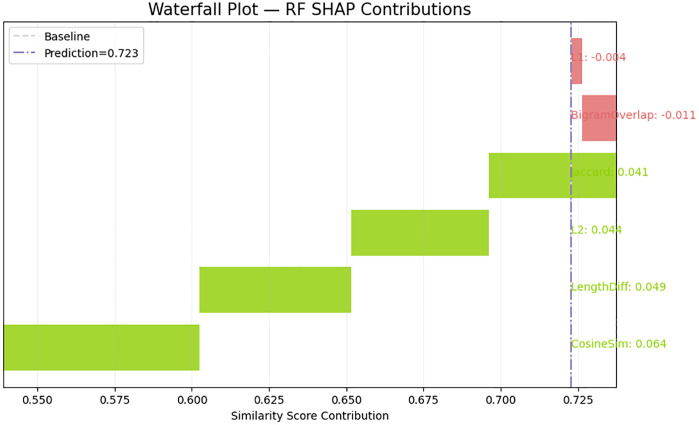
Local SHAP breakdown illustrating how individual features raise or lower the predicted similarity score relative to the model’s performance.

**Fig 17:**
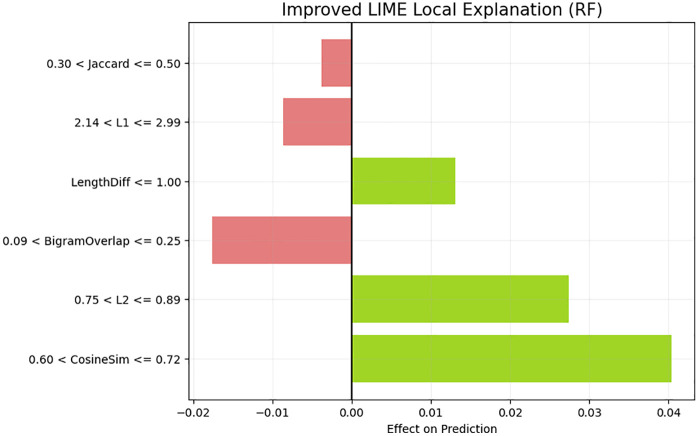
LIME Local Explanation for proposed model Interpretation showing the contribution of specific feature intervals to the model’s prediction.

The SHAP waterfall plot in Fig 16 gives localized consideration of a single prediction, which depicts how the individual features shift the final similarity score above or below the expectation at the baseline. Cosine similarity and L2 distance have the greatest ascending drifts that support their positive role observed across the world. Jaccard similarity also is of significant positive contribution, whereas Bigram Overlap and L1 distance slightly lower the prediction, which means that in the case of this specific sentence pair, the structural or phrase-level discrepancies negate to some degree the semantic similarity in the embedding-based features. This localized explanation confirms the joint influence of semantic and lexical clues on the influences of model output. The LIME local explanation in Fig 17 supplements the SHAP output, as it determines special intervals of features that contributed to the prediction of the model. The similarity score being predicted by high cosine similarity and moderate L2 norm is higher whereas the similarity score being predicted by higher L1 distance and moderate bigram mismatch is lower. In comparison to SHAP, which breaks down the prediction additively, LIME draws attention to the feature thresholds and interpretable conditions (e.g., 0.75 < L2 0.89). Altogether, the XAI outcomes validate the hypothesis that the similarity model relies mostly on the semantic proximity and tamps the similarity signal with structural dissimilarity between sentences.

### 4.2 Comparative analysis using all models

**Fig 18:**
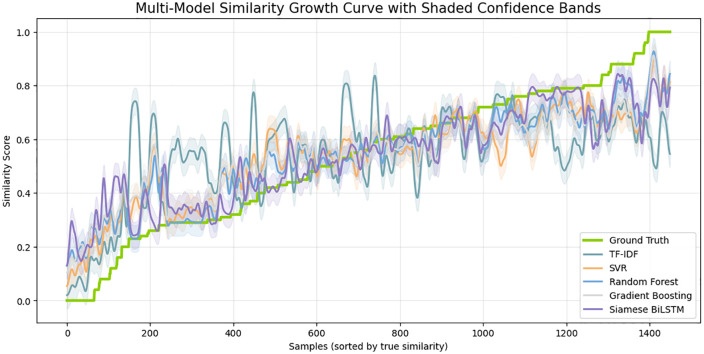
Comparative analysis of similarity score progression of all models against the ground truth, with shaded uncertainty regions.

**Fig 19:**
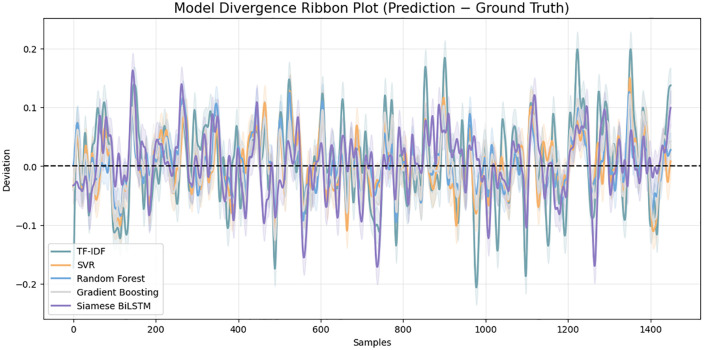
Comparison based on per-sample prediction deviation for all models. The Siamese BiLSTM exhibits the smallest residual fluctuations, indicating higher prediction stability and resilience to lexical inconsistency.

**Fig 20:**
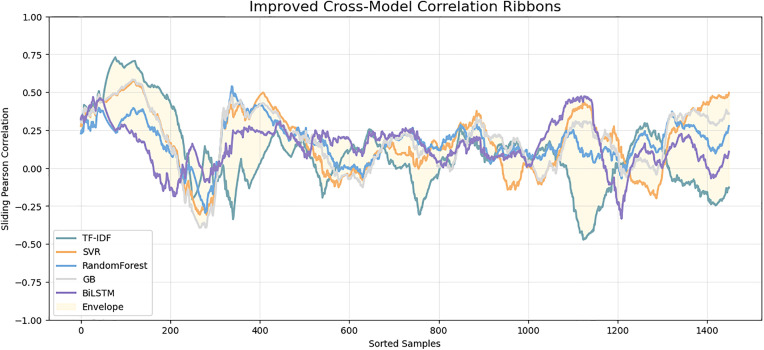
Sliding-window Pearson correlation trends comparing models across sorted test samples. The envelope band shows the overall correlation stability range, highlighting the enhanced consistency and semantic robustness of the BiLSTM model.

Fig 18 above presents the multi-model similarity growth curve which gives a holistic comparative analysis of how each model recapitulates the progression of semantic similarity when samples are ranked in an ascending order of ground-truth values. Both baseline TF-IDF and SVR show a high variance and spikes, which means that the two are unstable and prone to lexical anomalies. Random Forest and Gradient Boosting decrease the magnitude of oscillations, yet they still demonstrate significant deviation off the ground-truth curve. On the other hand, the Siamese BiLSTM curve adheres to the semantic flow more closely, and it has a smoother curve with the ground truth being followed with little error. The divergence ribbon plot in Fig 19 provides more insight into the reliability of the model since it visualizes the prediction differences to the ground truth. All the models will have variations around the zero line, but classical baselines, in particular, TF-IDF and SVR, will exhibit larger and more repeated divergence spikes. Such deviations reflect poor semantic correspondence and prone to length imbalance, token alignment and structural variation. Models that are based on trees decrease the level of divergence, though the trends are sparse. Comparatively, the Siamese BiLSTM has the most stable deviation distribution with the residual values concentrated around zero. Such small shapes illustrate that the model is skilled in capturing underlying semantic representations and ability to withstand noise in the input text. The ribbons on the cross-model correlation in Fig 20 show the consistency of each model with the ground-truth similarity trend among the ranked test samples. The TF-IDF and SVR have high levels of fluctuation which means that they are sensitive to sentence-level variations and cannot generalize semantically. RF and GB demonstrate a smoother correlation, but still, there is visible drift in the areas with a high linguistic complexity.

**Fig 21:**
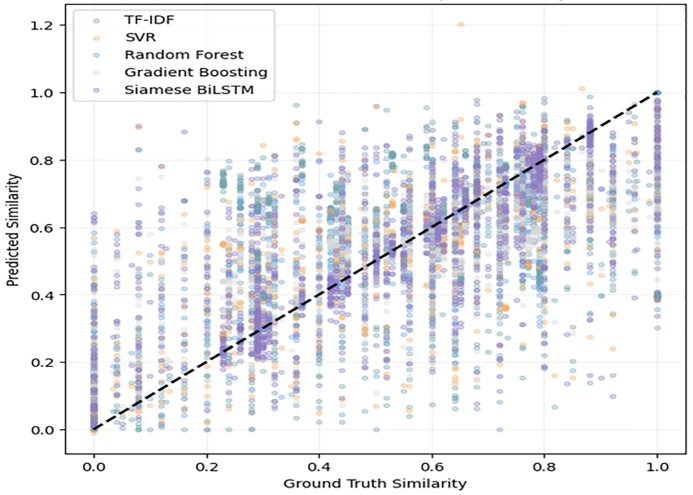
Scatterplot showing predicted similarity versus ground-truth similarity for all models.

Conversely, the Siamese BiLSTM has a less fluctuating correlation pattern particularly at the mid- and high-similarity levels indicating that it can retain semantical coherence through different sentence lengths and structures. The shaded envelope indicates the general stability range where the BiLSTM is always nearer to the center band than the classical models. Overall, the value validates the fact that deep semantic modeling provides more accurate similarity alignment across the dataset. The overall comparison of the accuracy of prediction is also demonstrated in the model calibration plot shown in Fig 21. In this case, the well-calibrated predictions are represented by scatter concentrations along the diagonal line.

The TF-IDF predictions are widely spread with low and medium similarities, which indicates its poor capacity of mapping lexical overlaps to actual semantic similarity. The clustering of SVR, Random Forest and Gradient Boosting is better; however, they are still widely scattered around the diagonal, which means that they are partially mis calibrated. Contrastingly, the Siamese BiLSTM exhibits high concentration of points in the diagonal line, especially in mid-to-high similarity areas. It means that the proposed deep semantic method does not only predict correct similarity values but also monotonic ordering which is also assessed in its Pearson and Spearman correlations. These findings confirm that deep contextual encoding is essentially more appropriate in the representation of subtle semantic similarity than shallow lexical or feature-based strategies.

The comparative performance results of Table 6 underline the effectiveness of the suggested Siamese Bi-LSTM framework with the aid of TF-IDF, cosine similarity and GloVe embeddings. Some of the previous methods like Re-LSTM with attention mechanisms and translation-based Siamese LSTM models had high MAE scores (4.2 and 2.3) which show suboptimal error performance. On the same note, machine learning regressors-based models, such as the Random Forest and XGB, and Bi-LSTM models with multi-embedding fusion obtained moderate Pearson correlation coefficients (r = 0.54 and r = 0.63, respectively), indicating a weak linear fit to ground truth similarity. On the contrary, the Pearson correlation of r = 0.76 in the proposed model is significantly higher, and it demonstrates greater consistency in prediction. The hybrid lexical and semantic features that are incorporated complement the quality of the representation and alignment and have shown to be more predictive for any solution than the existing solutions.

**Table 6:** Comparison analysis of existing approaches performance with proposed study.

Reference	Year	Model	Technique	Dataset	Results
[28]	2022	Re-LSTM	Weighted embedding + attention	Text embeddings	MAE: 4.2
[32]	2024	Siamese Bi-LSTM	Translation augmentation + LSTM	Semantic Similarity Kaggle	MAE: 2.3
[33]	2025	Random Forest, XGB	Predict sim from embed features	Semantic Similarity Kaggle	r = 0.54
[24]	2026	Bi-LSTM	Multi-embedding (GloVe, FT, Para)	Custom dataset	r = 0.63
Proposed	**Siamese Bi-LSTM**		**TF-IDF, Cosine Sim, GloVe**	**Semantic Similarity Kaggle**	**r = 0.76** **MAE: 0.107**

## 5. Conclusion and future work

This study examined the effectiveness of deep semantic representations for sentence-level similarity prediction and demonstrated the clear advantages of adopting neural architectures over traditional lexical approaches. By employing a Siamese BiLSTM framework, the model was able to capture contextual meaning, structural variations, and paraphrastic relationships that shallow similarity measures fail to represent. The results consistently showed that deep contextual embeddings provide a more accurate and linguistically grounded interpretation of similarity, enabling the model to generalize effectively across diverse sentence constructions. These findings reinforce the importance of semantic representation learning as a core component of modern natural language understanding. Feature-level analysis further revealed that lexical similarity measures capture only shallow overlaps, whereas embedding-based distances encode deeper semantic structure, explaining the superior generalization of the proposed model. Overall, the results confirm that contextualized representation learning is well-suited for semantic similarity tasks that require nuanced understanding beyond surface form. Future research may explore transformer-based Siamese architecture, domain-adaptive training strategies, and cross-lingual semantic modeling to further enhance generalization and expand applicability across broader linguistic contexts. Moving onward, future work will expand to explore transformer-based Siamese architectures such as fine-tuning Sentence-BERT or integrating contrastive learning frameworks like SimCSE. These models offer the potential to capture deeper contextual semantics and improve similarity prediction in low-resource or domain-specific settings.
